# Melatonin Positively Influences the Photosynthetic Machinery and Antioxidant System of *Avena sativa* during Salinity Stress

**DOI:** 10.3390/plants8120610

**Published:** 2019-12-16

**Authors:** Nisha Varghese, Onoud Alyammahi, Sarah Nasreddine, Abla Alhassani, Mayank Anand Gururani

**Affiliations:** Department of Biology, College of Science, United Arab Emirates University, Al Ain P.O. Box 15551, UAE; nishavarghese@uaeu.ac.ae (N.V.); 201300708@uaeu.ac.ae (O.A.); 201735162@uaeu.ac.ae (S.N.); 201706863@uaeu.ac.ae (A.A.)

**Keywords:** abiotic stress, chlorophyll-a, fluorescence, melatonin, photosystem II, quantitative PCR, reactive oxygen species

## Abstract

Recent studies have demonstrated melatonin protects various crops against abiotic stresses. However, the effects of melatonin on the photosynthetic apparatus of stressed plants is poorly characterized. We investigated the effects of melatonin pretreatment on photosynthesis and tolerance to salinity stress in *Avena sativa* (oat) plants. Oat plants were exposed to four treatments (three replicate pots per treatment): well-watered (WW; control); watered with 300 mM salt solution for 10 days (NaCl); pretreated with 100 µM melatonin solution for 7 days then watered normally for 10 days (Mel+W); or pretreated with 100 µM melatonin for 7 days then 300 mM salt for 10 days (Mel+NaCl). Considerable differences in growth parameters, chlorophyll content, stomatal conductance, proline accumulation, lipid peroxidation, electrolyte leakage, and growth parameters were observed between groups. Genes encoding three major antioxidant enzymes were upregulated in the Mel+NaCl group compared to the other groups. Chlorophyll-a fluorescence kinetic analyses revealed that almost all photosynthetic parameters were improved in Mel+NaCl plants compared to the other treatments. Analysis of genes encoding the major extrinsic proteins of photosystem II (PSII) revealed that PsbA, PsbB, PsbC, and PsbD (but not PsbO) were highly upregulated in Mel+NaCl plants compared to the other groups, indicating melatonin positively influenced photosynthesis under control conditions and salt stress. In addition, melatonin upregulated stress-responsive NAC transcription factor genes in plants exposed to salt stress. These findings suggest melatonin pretreatment improves photosynthesis and enhances salt tolerance in oat plants.

## 1. Introduction

Despite significant efforts and advancements in abiotic stress research, soil salinity remains one of the most detrimental factors in agriculture and affects approximately 20% of irrigated land [1]. The complex, intricate physiological responses of plants to salinity stress make it difficult to assess the physiological, biochemical, and molecular impacts of salinity. At a basic level, salinity suppresses water uptake as the excess salts present in the soil or irrigation water reduce absorption of water by plants. Salinity also induces oxidative stress, characterized by the accumulation of reactive oxygen species (ROS), which cause multi-faceted damage to plant cells ranging from inhibition of photosynthesis [2] to electrolyte leakage, lipid peroxidation, and damage to nucleic acids [3]. Plants have evolved mechanisms to overcome ROS-induced damage by producing increased levels of water-soluble antioxidants, such as ascorbate, lipid-soluble antioxidants, including α-tocopherol, and ROS-scavenging antioxidant enzymes, such as superoxide dismutase (SOD), catalase (CAT), and ascorbate peroxidase (APX) [3,4]. In addition, to better understand the mechanisms underlying the plant antioxidant system, it is imperative for plant researchers to identify compounds that may potentially induce stress tolerance in plants. Recent reports have indicated that the application of melatonin alleviated a variety of abiotic stresses in different crops [5,6,7], indicating that melatonin could be a promising compound to prevent abiotic stress. 

Melatonin (N-acetyl-5-methoxytryptamine) is a natural antioxidant found in all living organisms, from bacteria to plants and animals. Melatonin production varies significantly in plant species [8]. Melatonin is synthesized from tryptophan via a series of reactions catalyzed by tryptophan decarboxylase, tryptamine 5-hydroxylase, serotonin N-acetyltransferase, and arylalkylamine N-acetyltransferase [8]. The genes encoding these enzymes have been characterized in a variety of plants and algae [9,10]. Melatonin is closely associated with ROS production and stress-related cell signaling, thus chloroplasts are presumed to be the major site of melatonin production [5]. Melatonin has higher antioxidant capacity than other non-enzymatic antioxidants, such as ascorbates and tocopherols, which may be related to the ability of melatonin to be efficiently transported through different cell compartments [5,11]. 

Numerous reports have indicated the beneficial ability of melatonin application to alleviate heavy metal stress [12], UV stress [13], salinity stress [14,15,16], drought stress, and high/low temperature stress [17,18] in various plant species. Recent studies suggest that exogenous application of melatonin in plants under salt stress leads to an increase in endogenous melatonin levels, partly via the phyto-melatonin receptor CAND2/PMTR1. Elevated melatonin levels then improve photosynthesis, ion homeostasis, and initiate a signaling cascade to boost the biosynthesis of hormones, nitric oxide, and polyamine (reviewed in [19,20]). Though the ability of melatonin to increase the tolerance of plants to different stresses is well documented, the precise mechanisms by which melatonin confers stress resistance are poorly characterized. Moreover, most studies have focused on the ROS scavenging capacity of melatonin; few studies have investigated the effects of melatonin on the photosynthetic machinery, especially under salinity stress. However, very recently, Yin et al. [21] have demonstrated the positive effects of melatonin in tomato plants under salt stress. It was found that pre-treatment of melatonin suppressed the production of ROS by establishing a consistent flow of electrons, improving the repair of photosystem II (PSII) by maintaining the abundance of PsbO and D1, and facilitating the ability of the donor and acceptor sides of PSII to deliver electrons.

Oats (*Avena sativa* L.) are one of the most important cereal and forage crops in the world, with an approximate global yield of 700,000 tons [22]. Oats have attracted significant attention as a healthy food in recent years as they contain soluble fibers with hypocholesterolemic properties. Salinity stress is a major constraint that limits the productivity of oats worldwide. Although oats are moderately salt tolerant compared to other major cereal crops, such as barley and wheat, salinity inhibits oat seed germination and hampers oat plant growth and development [23].

In this study, we examined the effects of melatonin on the growth, antioxidant defense system, biochemical indicators, expression of genes encoding antioxidant enzymes, proline synthesis, and photosynthesis in oat plants exposed to high salinity stress. We demonstrated that pretreatment with 100 µM melatonin substantially promoted growth, improved the antioxidant system, and protected the photosynthetic machinery in oat plants under salt stress.

## 2. Results

### 2.1. Melatonin Attenuates Morphological and Physiological Changes in Oat Plants Exposed to Salt Stress

**Figure 1:**
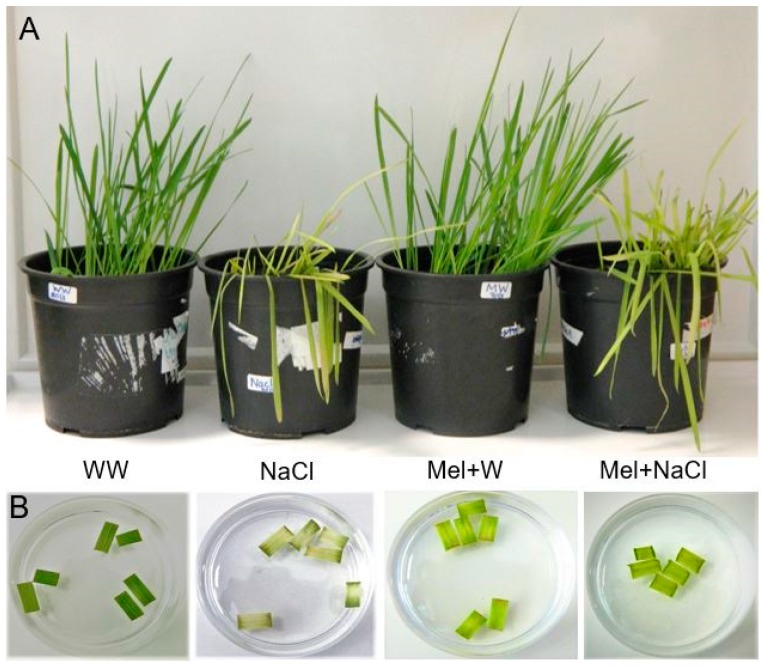
(**A**) Effect of pretreatment with melatonin on morphological differences between four treatments in oat plants. (**B**) Leaf disc senescence assay in oat plants. WW, well-watered; NaCl, plants exposed to 300 mM NaCl; Mel+W, plants treated with melatonin and water; Mel+NaCl, plants exposed to 300 mM NaCl and treated with melatonin.

Marked differences were observed in the morphology of each group of plants on day 17 (Figure 1A). The plants in the WW control group (exposed to water only) and the Mel+W group pretreated with melatonin appeared normal. The NaCl group exposed to 300 mM NaCl for 10 days showed clear signs of stress as their leaves had started to turn brown. However, the Mel+NaCl group pretreated with melatonin for 7 days and then exposed to 300 mM NaCl for 10 days exhibited considerably better growth than the NaCl group exposed to NaCl without melatonin pretreatment. 

The growth parameters reflected the obvious morphological differences between groups. Both WW and Mel+W plants exhibited notably better growth in terms of shoot and root length and fresh weight compared to the Mel+NaCl and NaCl plants. However, the Mel+NaCl group had a remarkably higher average root length, shoot length, and fresh weight than the NaCl group (Table 1). 

The leaf disc senescence bioassay was performed to estimate the effect of melatonin on salt tolerance in oat plants (Figure 1B). Leaf pieces from untreated plants were floated on water (control), 100 µM melatonin, 300 mM NaCl, or 100 µM melatonin +300 mM NaCl. Visible differences in the greenness of the pieces were observed 3 days later: the control leaves appeared dark green, whereas the leaves exposed to Mel+W and Mel+NaCl were a lighter green color. The leaves exposed to NaCl appeared extremely light green or pale yellow, indicating the harsh effects of NaCl-induced salt stress (Figure 1B). 

The total chlorophyll contents of the leaves exposed to WW and Mel+W were similar. The total chlorophyll content of the leaves exposed to WW and Mel+W were almost 1.6-fold higher than the leaves exposed to NaCl. However, the Mel+NaCl leaves accumulated substantially higher chlorophyll contents than the leaves exposed to NaCl alone (Table 1). 

### 2.2. Melatonin Increases the Proline Content, Reduces the Malondialdehyde (MDA) Content and Electrolyte Leakage, and Improves Stomatal Conductance in Oat Plants under Salt Stress

**Figure 2:**
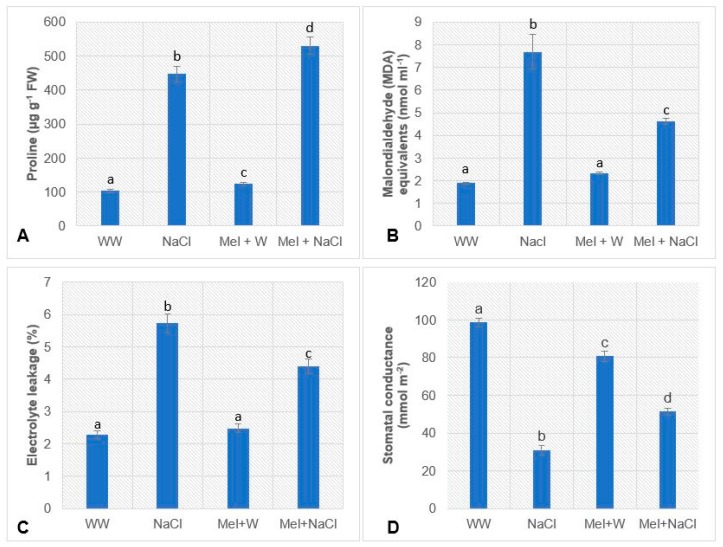
Estimation of (**A**) proline content, (**B**) MDA content, (**C**) electrolyte leakage, and (**D**) stomatal conductance in oat plants. WW, well-watered; NaCl, plants exposed to 300 mM NaCl; Mel+W, plants treated with melatonin and water; Mel+NaCl, plants exposed to 300 mM NaCl and treated with melatonin. Different letters in each column indicate significant differences (*p* ≤ 0.05) in between treatments after Tukey’s test (*n* = 3).

The proline contents of treated leaves harvested on day 17 were assayed to elucidate the effects of melatonin on proline biosynthesis in oat plants exposed to salinity stress (Figure 2A). The plants in the NaCl group exposed to 300 mM NaCl accumulated a substantially higher proline content than the WW and Mel+W groups. Interestingly, the Mel+NaCl group had a higher proline content than the NaCl group. 

The impact of NaCl-induced salinity stress on peroxidation of membrane lipids and membrane permeability were determined by estimating the MDA content and electrolyte leakage, respectively (Figure 2B,C). There was no significant difference in the MDA content of the WW and Mel+W groups, though the MDA content of the NaCl group was 4- and 3.2-fold higher than the WW and Mel+W groups, respectively. However, the Mel+NaCl group accumulated 1.6-fold lower levels of MDA than the NaCl group. 

Similarly, electrolyte leakage (%) was not significantly different between the WW and Mel+W groups, but was almost 2.5-fold higher in the NaCl group. However, the Mel+NaCl group exhibited substantially lower electrolyte leakage than the NaCl group. 

Highest stomatal conductance (98.6%) was recorded in the WW group, followed by the Mel+W group (80.8%). The NaCl group exposed to 300 mM NaCl had the lowest stomatal conductance. However, the Mel+NaCl (51.6%) group exhibited noticeably higher stomatal conductance than the NaCl group (31%; Figure 2D).

### 2.3. Melatonin Affects the Expression of Enzymes Involved in Proline Biosynthesis in Oat Plants under Salt Stress 

**Figure 3:**
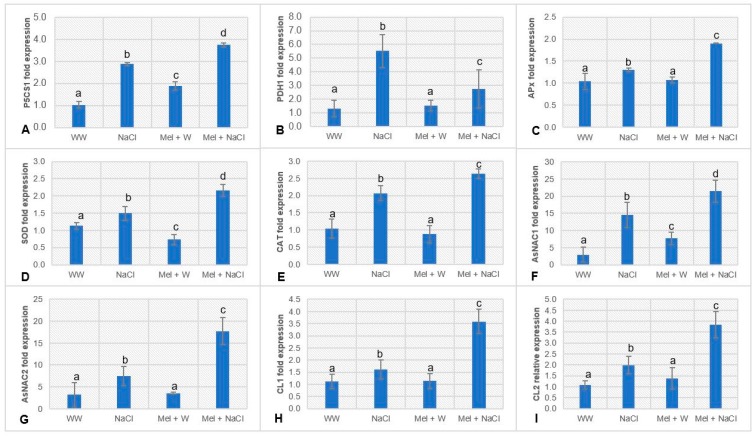
Real-time mRNA expression of genes encoding (**A**) P5CS1 and (**B**) PDH1, associated with proline biosynthesis and genes encoding ROS-scavenging enzymes, (**C**) Ascorbate peroxidase (APx), (**D**) superoxide dismutase (SOD), and (**E**) catalase (CAT), and genes encoding stress-inducible (**F**) NAC1, (**G**) NAC2, (**H**) CL1, and (**I**) CL2 proteins in oat plants. Fold expression values are normalized to those of Actin control. WW, well-watered; NaCl, plants exposed to 300 mM NaCl; Mel+W, plants treated with melatonin + water; Mel+NaCl, plants exposed to 300 mM NaCl and treated with melatonin. Different letters in each column indicate significant differences (*p* ≤ 0.05) in relative gene expression between treatments after Tukey’s test (*n* = 6).

Expression of pyrroline-5-carboxylate synthetase 1 (P5CS1, an enzyme related to proline biosynthesis) and proline dehydrogenase 1 (PDH1, involved in proline breakdown) were determined on day 17 (Figure 3A,B). P5CS1 expression was markedly higher in the NaCl group than the WW and Mel+W groups. However, the Mel+NaCl group expressed 1.3-fold higher levels of P5CS1 compared to the NaCl group. The PDH1 gene also exhibited a clear response to salt stress: PDH1 was expressed at the highest levels in the NaCl group. The Mel+NaCl group expressed substantially higher levels of PDH1 compared to the Mel+W and WW groups, but far lower levels than the NaCl group.

### 2.4. Melatonin Upregulates ROS Scavenging Enzymes in Oat Plants under Salt Stress

APx, SOD, and CAT gene expression were compared on day 17 (Figure 3C–E). There were no significant differences in the expression of these genes between the WW and Mel+W groups. However, great differences were observed between the NaCl and Mel+NaCl groups: Mel+NaCl plants exhibited 1.4-, 1.4-, and 1.2-fold higher levels of APx, SOD and CAT expression, respectively, compared to the NaCl group.

### 2.5. Melatonin Upregulates Stress-Inducible NAC Genes in Oat Plants under Salt Stress

We also profiled the expression of four genes randomly selected from a panel of previously reported stress-inducible NAC genes [24]. The expression of NAC1, NAC2, CL1, and CL2 were not significantly different between the WW and Mel+W groups (Figure 3F–I). However, NAC1 and NAC2 expression were markedly higher in the NaCl group than in the WW and Mel+W groups. Interestingly, the Mel+NaCl group exhibited far higher levels of NAC1 and NAC2 than the NaCl group (1.4- and 2.3-fold increases, respectively). Similarly, the NaCl group expressed much higher levels of CL1 and CL2 compared to the WW and Mel+W groups. However, the Mel+NaCl group expressed 2.2- and 1.9-fold higher levels of CL1 and CL2, respectively, compared to the NaCl group. 

### 2.6. Melatonin Enhances Chlorophyll-a Fluorescence Kinetics in Oat Plants under Salt Stress

**Figure 4:**
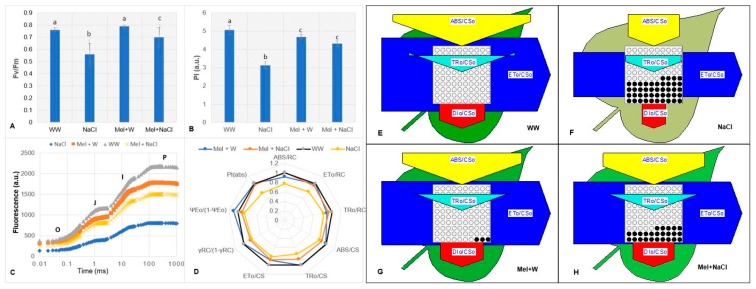
Chlorophyll-a fluorescence kinetics of the OJIP curve of dark-adapted leaves of oat plants. (**A**) Estimation of photochemical efficiency in terms of Fv/Fm and (**B**) estimation of performance index (PI) in dark-adapted leaves of oat plants. (**C**) Transient curves of each line represent the average of 15 measurements per treatment. (**D**) Radar plot with a series of parameters derived from JIP-test analyses of the experimental fluorescence OJIP transients. (**E**–**H**) Energy pipeline leaf model of phenomenological fluxes (per cross-section, Cso) in (**E**) well-watered (WW) oat plants, (**F**) oat plants exposed to salinity stress induced by 300 mM NaCl, (**G**) oat plants treated with melatonin and watered normally (Mel+W), and (**H**) oat plats treated with melatonin and exposed to salinity stress induced by 300 mM NaCl (Mel+NaCl). The value of each parameter can be seen in relative changes in width of each arrow. Active reactive centers (RCs) are shown as open circles and inactive RCs are closed circles. Different letters in each column in (**C**) and (**D**) indicate significant differences (*p* ≤ 0.05) in between treatments after Tukey’s test (*n* = 9). WW, well-watered; NaCl, plants exposed to 300 mM NaCl; Mel+W, plants treated with melatonin and water; Mel+NaCl, plants exposed to 300 mM NaCl and treated with melatonin.

Damage to PSII reaction centers (RCs) can be assessed by measuring maximal photochemical efficiency (Fv/Fm), which indicates a plant’s ability to grow under unfavorable environmental conditions [25]. The NaCl and Mel+NaCl groups had remarkably lower Fv/Fm ratios than the WW and Mel+W groups. However, the NaCl group had a lower Fv/Fm ratio (0.57) than the Mel+NaCl group (0.69; Figure 4A). 

The performance index (PI) is an indicator of plant vitality and is associated with the overall outcome of plant growth or survival under stress conditions, such as salinity, drought, or high/low temperatures [26]. The plants exposed to NaCl alone had much lower PI values than the WW, Mel+W, and Mel+NaCl groups (Figure 4B). The chlorophyll-a fluorescence transients of the dark-adapted oat plants exhibited the typical OJIP shape when plotted on a logarithmic scale from 20 μs to 1 s (Figure 4C), implying all of the plants were photosynthetically active. Notably, the NaCl group had lower J, I, and P peaks at all time points, resulting in a remarkably different OJIP curve compared to the WW, Mel+W, and Mel+NaCl groups. 

The fluorescence transients were further reduced to eight major structural and functional photosynthetic parameters. The average values of these parameters in the NaCl, Mel+W, and Mel+NaCl groups were normalized to the values for the WW control group and plotted as a radar plot (Figure 4D). Thus, the variations indicate the impact of each treatment relative to WW. The ABS/RC parameter indicates the ratio of total absorption of PSII antenna chlorophylls to the number of active RCs. ETo/RC represents photosynthetic electron transport per active RC of PSII. The phenomenological energy fluxes per leaf cross-section (CS) for absorption (ABS/CS), trapping (TRo/CS), and electron transport (ETo/CS) were also determined [27,28]. The parameter γRC/(1−γRC) denotes the portion of Chl-a molecules that function as RCs, reflecting the portion of absorbed energy that reaches RCs. The capability to transfer excited electrons to the photosynthetic electron transport chain is represented by the parameter ψ0/(1 − ψ0). Finally, the photosynthetic performance index (PI_ABS_) serves as an indicator of three critical photosynthetic steps (ABS, TR, and ET) and, thus, reflects photosynthesis overall. All of these parameters were lower in the NaCl group than the WW and Mel+W groups, but were higher in the Mel+NaCl group than in the NaCl group, indicating melatonin treatment positively influenced photosynthesis during salt stress.

### 2.7. Melatonin Attenuates the Reductions in Energy Pipeline Model Parameters in Oat Plants under Salt Stress

The parameters ABS/CS0, TR0/CS0, ET0/CS0 and DI0/CS0 were analyzed via the energy pipeline leaf model of phenomenological fluxes using Biolyzer 4HP software (Figure 4E–H). This model typically provides information on the flow of energy from the chlorophyll antenna molecules to the photosynthetic electron transport chain via the RCs of PSII [29,30]. The capability of the plants in each group for light absorption (ABS/CS0), trapping, electron transport (TR0/CS0), and dissipation per cross-section of PSII (DI0/CS0) are indicated by the sizes of the arrows in Figure 4. The impact of salinity stress on the plants in the NaCl group, which was reflected by a visible reduction in the greenness of the leaves (Figure 1), was primarily due to a large number of inactive RCs (shown as closed circles in Figure 4F). Similarly, the differences in the sizes of arrows representing each parameter indicate the negative impact of 300 mM NaCl on the photosynthetic apparatus in the NaCl group. In contrast, these arrows were larger for the Mel+NaCl group than the NaCl group (Figure 4H), indicating a positive correlation between melatonin treatment and the photosynthetic parameters ABS/CSo, TR/CSo, ETo/Cso, and DIo/CSo.

### 2.8. Melatonin Upregulates Genes Encoding Core PSII Proteins in Oat Plants under Salt Stress

**Figure 5:**
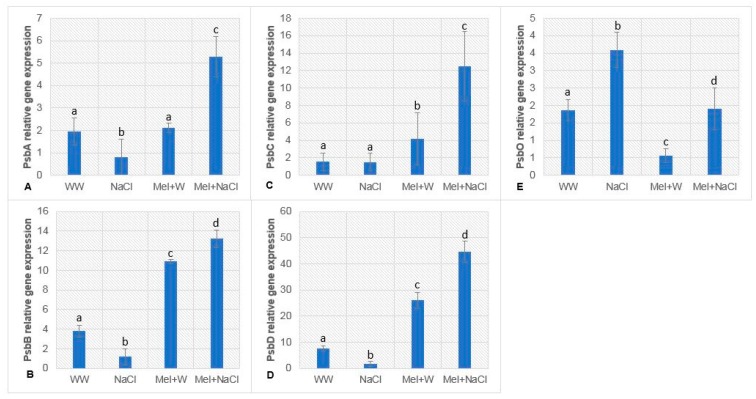
Real-time mRNA expression of genes encoding photosystem II-associated proteins. (**A**) PsbA, (**B**) PsbB, (**C**) PsbC, (**D**) PsbD, and (**E**) PsbO in oat plants. Fold expression values are normalized to those of actin control. WW, well-watered; NaCl, plants exposed to 300 mM NaCl; Mel+W, plants treated with melatonin and water; Mel+NaCl, plants exposed to 300 mM NaCl and treated with melatonin. Different letters in each column indicate significant differences (*p* ≤ 0.05) in relative gene expression between treatments after Tukey’s test (*n* = 6).

In order to assess the putative effects of melatonin on the core PSII proteins during salinity stress, qPCR was performed to determine the expression of PsbA (which encodes the D1 subunit of PSII), PsbB (CP47 subunit of PSII), PsbC (CP43), PsbD (D2 subunit of PSII), and PsbO (33 kDa oxygen-evolving complex of PSII). As shown in Figure 5A–E, NaCl suppressed the expression of the *PsbA*, *PsbB*, and *PsbD* genes, but not *PsbC*, compared to the WW group. However, the expression of all four genes was remarkably higher in the Mel+NaCl group compared to the NaCl group. Interestingly, PsbO was expressed at substantially higher levels in the NaCl group than the other groups. The expression of PsbO in NaCl plants was approximately 2-fold higher compared to the Mel+NaCl and WW groups and 4-fold higher compared to the Mel+W groups. 

## 3. Discussion

We examined the effects of melatonin on the photosynthetic components of a higher plant under salinity stress. In addition to ameliorating the symptoms of salinity stress, melatonin greatly reduced various stress-induced changes in parameters that determine the efficiency of primary PSII photochemistry. Our results also indicated that pretreatment with melatonin altered the expression of core proteins that constitute the PSII supercomplex, under both normal and salinity stress conditions. 

Assessment of morphological differences after 10 days of stress treatment (Figure 1A) indicated melatonin exerted positive effects under both normal and stress conditions (Table 1). Melatonin increased the chlorophyll content (Figure 1B; Table 1) and proline accumulation (Figure 2A), reduced the levels of MDA (Figure 2B) and electrolyte leakage (Figure 2C), and increased stomatal conductance (Figure 2D), in accordance with previous reports [14,15,31,32]. Proline plays multiple roles in higher plants, including maintenance of membrane integrity, removal of ROS, regulation of osmotic pressure, and acting as an osmoprotectant against a range of abiotic stresses, including high salinity [33]. An increase in the endogenous proline content is known to confer abiotic stress tolerance and is associated with upregulation of the *P5CS1* gene and downregulation of the *PDH* gene [34,35]. P5CS1 is involved in the first step of proline synthesis and catalyzes the conversion of *L*-glutamic acid to glutamate*-5-*semialdehyde [33]. Previous studies demonstrated that overexpression of *P5CS1* can ameliorate salinity stress in economically important crops like rice and potato [36,37]. On the other hand, abiotic stress tolerance has been associated with lower *PDH1* gene expression [22]. We observed appreciably enhanced accumulation of proline, accompanied by a 1.3-fold increase in P5CS1 transcript levels and 2-fold reduction in PDH1 transcript levels in Mel+NaCl plants compared to NaCl plants (Figure 3A,B). This implies that melatonin positively influences proline accumulation during salinity stress in higher plants. 

Adverse environmental conditions, such as drought, heat, and salinity, induce the generation of toxic ROS molecules that consequently lead to membrane lipid peroxidation [3]. ROS scavenging antioxidant enzymes, such as APX, CAT, and SOD, eliminate toxic free radicals from cells. To illustrate the role of the antioxidant machinery during salinity stress in oat plants treated with or without melatonin, we performed real-time PCR analysis of genes encoding the major antioxidant enzymes in higher plants (Figure 3C–E). The APx, SOD, and CAT genes were upregulated in the Mel+NaCl group compared to the NaCl group, in accordance with previous studies [38,39]. Foliar application of melatonin along with cold priming has been reported to have positive effects on photosynthesis, stomatal conductance, antioxidant enzyme activity, and expression of related genes in wheat under cold stress [40]. Similarly, melatonin increased the activity of antioxidant enzymes and promoted the accumulation of antioxidants in wheat seedlings subjected to cold stress [41]. Another recent report suggested that melatonin alleviated cadmium stress in wheat seedlings by increasing the activity of the key antioxidant enzymes APX, SOD, CAT, and POD (peroxidase) [42]. 

Next, we performed real-time PCR analysis of NAC proteins that were recently reported to be induced during the onset of salinity stress in oat plants [24]. These stress-inducible NAC genes have been exploited to genetically engineer abiotic stress-tolerant crops [43]. NAC1, NAC2, CL1, and CL2 were expressed at 1.5-, 2.3-, 2.2-, and 1.9-fold higher levels, respectively, in Mel+NaCl plants compared to NaCl plants (Figure 3F–I). These findings clearly indicate melatonin positively increased the tolerance of oat plants to salinity. 

The impact of abiotic stresses on the photosynthetic machinery have been studied in detail using chlorophyll-a fluorescence kinetics [2,44,45,46,47]. This method has been used extensively to study the impact of various abiotic stresses on the photosynthetic machinery of various crop plants. Recent work by Kalaji et al. (2018) demonstrated that, although the parameters used in these analyses cannot be used as indicators to distinguish between different stresses, the response of photosystem II to applied stress occurred earlier in drought-stressed plants compared to salinity-stressed plants [48]. Fast fluorescence induction is comprised of four phases: O, the origin, P, the peak, and J–I, the intermediate phases [49,50]. Fast induction kinetics reflect the primary photochemistry of PSII and provide critical information on the reduction of electron acceptors in the electron transport chain (ETC) [2]. The parameter Fv/Fm indicates whether abiotic stress affects PSII II. Salt-stressed barley plants were reported to have a drastic effect on the Fv/Fm values as this value was only 25% in stressed plants compared to that in control plants, indicating that the RCs were photochemically inactive in the stressed plants [51]. The Fv/Fm ratio was markedly higher in Mel+NaCl plants than NaCl plants, further indicating that melatonin had a positive effect in plants exposed to salinity stress (Figure 4A). The performance index (PI) is an indicator of sample “vitality” and is closely related to plant growth and survival under adverse environmental conditions [50]. The PI was almost 1.6-fold higher in Mel+NaCl plants than in NaCl plants, which reflected the improved growth parameters and overall greener and healthier appearance of the Mel+NaCl plants (Figure 4B). 

Exposure to 300 mM NaCl drastically affected the OJIP transient of oat plants in the O–J, J–I, and the I–P phases (Figure 4C, blue line); however, pre-treatment with melatonin attenuated these changes. Previous reports have suggested a decline in the transient polyphasic curves is related to inhibition of ETC at the donor site of PSII, along with a decrease in the quinone A pool, the electron acceptor in PSII [27,52]. Thus, these results indicated that pretreatment with melatonin attenuated the negative effects of NaCl-induced salinity stress on the ETC.

The JIP test converts Chl-a fluorescence data to biophysical parameters that quantify the energy flow through PSII [27,53,54]. In addition to evaluating the damage to the acceptor side of PSII, OJIP analysis also calculates the crucial parameters ABS, TR, ET, and RE [27,50]. Energy fluxes per RC is a specific functional parameter, while the energy fluxes per excited cross section (CS) indicates the corresponding phenomenological energy fluxes. It is well documented that salinity induces a negative effect on the quantum yield of PSII electron transport, the energy available for the reaction centers, and the efficiency of oxygen evolving complex (OEC) [51]. In another report, two weeks of salinity stress resulted in a 76% decrease in quantum efficiency of PSII in *Arabidopsis* plants [54]. We noted that NaCl stress greatly reduced ABS/RC, ET0/RC, and TR0/RC compared to oat plants under non-stress conditions (Mel+W) or plants exposed to salinity after pretreatment with melatonin (Mel+NaCl; Figure 4D). The higher average ABS, TR, and ET values of Mel+NaCl plants due to inactivation of some RCs suggests that a decrease in the size of the functional antennae in salt-stressed plants accelerated energy transfer to active RCs. This inference is in accordance with previous reports that leaves under stress continuously regulate electron flux by reducing the PSII antenna size and increasing controlled dissipation of energy [27,55]. Under both normal and salt stress conditions, melatonin-treated plants, i.e., Mel+W and Mel+NaCl plants, exhibited higher specific fluxes values, indicating melatonin increased the efficiency of energy utilization in both normal and NaCl-stressed plants. The increased phenomenological fluxes (ABS/CS, TR0/CS, and ET0/CS) of Mel+NaCl plants led to a notable increase in the PI(abs), which facilitates the energy cascade from the first absorption events to plastoquinone reduction [4,27]. The energy pipeline leaf model can reveal critical detail on the efficiency of energy flow from antennae to ETC components through the PSII reaction center. NaCl reduced the ABS/CS0, indicating reduced energy absorption per excited cross-section (Figure 4E–H). The parameter ET0/CS0 represents the ETC in a PSII cross-section and the rate at which quinone A (Q_A_) is reoxidized over a cross-section of active RCs. The higher ET0/CS0 of the NaCl group indicated inhibition of PSII RCs and the severe effects of NaCl stress on the PSII-donor side compared to Mel+NaCl plants, further confirming the protective role of melatonin in plants under salinity stress. Plants dissipate excess absorbed energy either as heat, known as non-photochemical quenching, or in the form of fluorescence and energy transfer to other systems [27]. The parameter DI0/CS0 denotes the total dissipation over the cross-section of active and inactive RCs. Interestingly, energy dissipation (DIo/RC) has been reported to increase under stress conditions because of the inactivation of some PSII RCs that leads to the protection of leaves from photo-oxidative damage as the excess energy is dissipated in the form of heat [56].The density of active RCs (Figure 4; open circles) reduced and the density of inactive RCs increased (Figure 4; dark circles) in both NaCl and Mel+NaCl plants under stress. However, this damage was much more severe in the NaCl group compared to the Mel+NaCl group. The improved photosynthetic parameters of Mel+W plants was an interesting observation and indicated that melatonin positively impacts the photosynthetic efficiency, even under non-stressed conditions. This finding is in line with the increased photosynthetic efficiency of *Chara australis* after exogenous application of melatonin [57]. Furthermore, our results suggest that melatonin helps to maintain PSII efficiency under abiotic stress, in agreement with other previous findings [7,8]. For instance, Wang et al. (2013) reported that—in addition to enhancing antioxidant enzyme activities—melatonin maintained the proper functioning of PSII in apple plants exposed to drought stress [58]. Melatonin also exerted beneficial effects on the ETC and other critical photosynthetic parameters in tomato seedlings exposed to salinity stress [59].

Melatonin modulates the expression of various genes, including those encoding redox network enzymes, photosynthetic proteins, and senescence-related proteins [7,8]. Transcriptomic analysis by Wei et al. (2015) revealed melatonin-treated soybean plants exposed to salt stress exhibited altered expression of genes involved in photosynthesis. Genes encoding extrinsic and intrinsic PSII proteins were suppressed by salt stress; however, these genes were upregulated in salt-stressed soybean plants pretreated with melatonin [60]. In this study, we performed qPCR to investigate the effect of melatonin on core PSII proteins in oat plants under salinity stress (Figure 5A–E). Various stresses are known to cause damage to the D1 protein (encoded by the *PsbA* gene) [2,47] and, as expected, we observed a substantially lower abundance of PsbA transcripts in NaCl-treated plants (Figure 5A). With the exception of *PsbO*, the other genes (i.e., *PsbA*, *PsbB*, *PsbC*, and *PsbD*) were greatly downregulated in the NaCl group compared to WW plants, but upregulated in melatonin-treated NaCl-stressed plants. These findings are consistent with previous findings (though PsbO was also upregulated in those studies) [60]. In addition to participating in the water splitting step that leads to release of oxygen, PsbO also plays a critical role in protecting PSII from photo-damage [61,62]. Antisense PsbO increased PSII activity for a short period and resulted in early tuberization in potato plants. Interestingly, the same potato plants exhibited improved photosynthetic parameters and antioxidant activities under abiotic stresses [26]. Yi et al. (2008) showed that RNAi-mediated silencing of PsbO inhibited the expression of PsbA and PsbC and led to a loss of variable fluorescence yield (Fv/Fm) [63], indicating a direct correlation between the expression of the *PsbO*, *PsbA*, and *PsbC* genes. These conclusions are in contrast to our findings, as PsbO transcript abundance increased in the NaCl group. One explanation could be the increased proline accumulation observed in NaCl-treated oat plants (Figure 2A). Previous studies have shown proline acts as an electron donor and may possibly compensate for the lack of a functional OEC [26,64,65]. Secondly, the variable expression of PsbO in different plants under stress could be associated with the varied numbers of PsbO isoforms in different plant species. Interestingly, Shi et al. [66] reported exogenous application of melatonin reduced photosynthetic processes, partially downregulated the genes encoding the 5, 10, and 11 kDa subunits of the PSII intrinsic proteins PsbO1 and PsbY, and improved abiotic stress resistance in Bermuda grass. 

**Figure 6:**
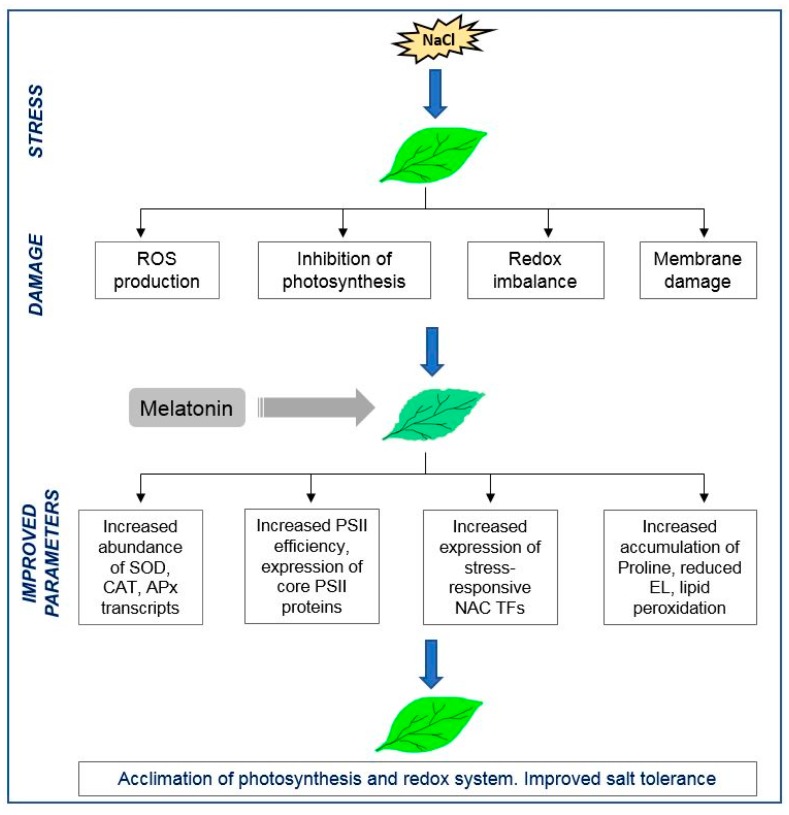
Hypothetical scheme of salinity stress tolerance with melatonin treatment in oat plants.

Taken together, our findings on the role of exogenous melatonin during salt stress can be summarized in a hypothetical scheme (Figure 6). Abiotic stress, such as high salinity, leads to the production of ROS that damage membranes, lipids, and DNA and initiate signaling cascades that eventually initiate signaling cascades that induce the expression of genes that that participate in the removal of ROS. Additionally, ROS molecules deteriorate the PSII assembly, leading to an imbalance in photosynthetic redox signaling, and inhibition of PSII repair. Melatonin has been shown to beneficially affect higher plants exposed to abiotic stresses by modulating the expression of genes encoding ROS scavenging enzymes, stress-responsive NAC transcription factors, and PSII core proteins. In addition, melatonin promoted the accumulation of higher levels of osmoprotectants, such as proline and stabilized membrane integrity, by reducing electrolyte leakage. Furthermore, melatonin may also act directly as an antioxidant towards ROS and lipid peroxides and, thus, ensures the survival of plants under salinity stress. 

## 4. Conclusions

In this work, we aimed to investigate the influence of melatonin application on the photosynthetic apparatus under salinity stress. We observed that melatonin positively and significantly influenced various morphological, physiological, and biochemical parameters in oat plants subjected to salinity stress. Furthermore, it was noted that melatonin also induced changes in the expression of genes that encode core PSII proteins. Based on our findings and analyses, we concluded that melatonin improves the PSII efficiency under salt stress by suppressing the stress-induced PSII damage and by promoting ROS scavenging by enzymatic antioxidants. It would be interesting to elucidate if the impact of melatonin on salt-stressed plants, as revealed in our findings, is directly associated with a change in the biosynthetic pathways of stress-responsive phytohormones. Given that melatonin exhibits positive regulatory role in plants, it is expected that melatonin could have potential applications in developing photosynthetically efficient stress tolerant transgenic crops in future. 

## 5. Methods

### 5.1. Plant Material and Experimental Design

Oat (*Avena sativa* L.) seeds purchased from the local market in Al Ain, UAE, were sown in pots (25 cm diameter) filled with soil peat and germination was observed over 3–4 days. Twelve pots of three-week-old, fully grown plants were selected and subjected to stress treatment, as previously described [67], with three pots/replicates for each of the four treatments: (1) the control well-watered (WW) group were irrigated with normal water for 10 days followed by 7 days irrigation with water; (2) the Mel+W group were pretreated with 100 µM melatonin in half-strength nutrient solution for 10 days, followed by normal irrigation for 7 days; (3) the Mel+NaCl group were pretreated with melatonin (100 µM) for 10 days, followed by 7 days irrigation with water containing 300 mM NaCl, and (4) the NaCl group were irrigated with normal water for 10 days, followed by 7 days irrigation with water containing 300 mM NaCl. The concentration of 100 µM melatonin was selected based on a recent report on the effect of melatonin on naked oat seedlings under drought stress [68]. 

Root length, shoot length, and plant fresh weight (FW) were recorded and the treated plants were subjected to analysis at the end of the experiment on day 17. Leaf samples were collected on day 17 and stored at −20 °C for other assays. Chlorophyll-a fluorescence was assessed on days 18, 19, and 20. The leaf senescence assay was conducted using untreated leaf samples.

### 5.2. Leaf Senescence Assay

The leaves from fully grown untreated oat plants were cut into pieces approximately 1.0 cm long and floated on 5 mL water (WW), 5 mL of 300 mm NaCl (NaCl), 5 mL of water supplemented with 100 µM melatonin (Mel + W), or 5 mL of 300 mm NaCl supplemented with 100 µM melatonin (Mel + NaCl). The leaf pieces were incubated in a plant growth chamber under a 16 h photoperiod at a light intensity of 100 µM m^−2^s^−1^ at 25 ± 1 °C until visual differences in greenness were observed between groups. The picture was taken after 3 days of experiment and then the chlorophyll content of the pieces was measured, as previously described [25]. 

### 5.3. Proline Estimation

Proline was extracted from frozen WW, Mel+W, NaCl, and Mel+NaCl leaves collected on day 17 and estimated colorimetrically, as previously described [4]. Approximately 500 mg frozen leaf samples were ground into a powder in liquid nitrogen and homogenized in 10 mL of 3% aqueous sulfosalicylic acid. Equal volumes (2 mL each) of the filtered homogenate, acid-ninhydrin, and glacial acetic acid were mixed and incubated for 1 h and the reaction was stopped by cooling the tubes on ice. The chromophore-containing phase was extracted with 4 mL of toluene and the absorbance of the extracts was measured at 520 nm. Proline concentration was determined using a standard curve constructed using known concentrations of proline and calculated on a fresh weight basis (µg g^−1^ FW).

### 5.4. Estimation of Malondialdehyde (MDA) Content 

The MDA content of frozen leaves collected on day 17 was estimated. Approximately 500 mg leaf tissue was ground to a powder in liquid nitrogen, homogenized in 5 mL of 50 mM buffer (0.07% NaH_2_PO_4_·2H_2_O and 1.6% Na_2_HPO_4_·12H_2_O), and centrifuged at 20,000 *g* for 25 min at 4 °C. Then, 4 mL of 20% trichloroacetic acid (TCA), containing 0.5% thiobarbituric acid (TBA), was added to 1 mL of the supernatant, incubated at 95 °C for 30 min, cooled on ice, centrifuged at 10,000 *g* for 10 min, and absorbance was determined at 532 nm. The value for non-specific absorption at 600 nm was subtracted from the absorbance reading at 532 nm. The concentration of MDA was calculated using an MDA extinction coefficient of 155 mM^−1^ cm^−1^ [69].

### 5.5. Stomatal Conductance 

Stomatal conductance was measured on the adaxial surface of fully developed intact leaves of intact plants on day 17 using a steady state diffusion leaf porometer (model SC-1; Decagon Devices, Inc., Pullman, WA, USA). The porometer was calibrated each day before the measurements and stomatal conductance was measured at 25 ± 1 °C and 55 ± 5% relative humidity.

### 5.6. Electrolyte Leakage

Electrolyte leakage was estimated on day 17 using the method described by Sullivan and Ross [70]. Leaf discs were boiled in a test tube containing 10 mL distilled water, the filtrate was collected, and electrical conductivity (EC) was measured and denoted EC_a_. The filtrate was heated to 55 °C for 30 min in a water bath, then electrical conductivity was measured again and denoted EC_b_. The filtrate was then boiled at 100 °C for 10 min and electrical conductivity was recorded and denoted EC_c_. Electrolyte leakage was calculated using:

Electrolyte leakage (%) = (EC_b_ − EC_a_ / EC_c_) × 100. (1)

### 5.7. Quantitative Real-Time PCR (qRT-PCR) Analysis

Total RNA was extracted from frozen leaves collected on day 17 using the Gene Jet RNA purification kit (Thermo Fisher, Waltham, MA, USA), treated with DNase I (Qiagen, Hilden, Germany), and cDNA was prepared from 1 μg RNA using the TruScript First Strand cDNA Synthesis Kit (Norgen, Canada) following the manufacturer’s instructions. The cDNA was diluted tenfold and used as a template for qRT-PCR analysis. The qRT-PCR was performed using QuantStudio 5 System (Applied Biosystems, Foster City, CA, USA) and EvaGreen qPCR System-ROX I (GeneDireX Inc., Taiwan). The primers are listed in Table S1. Glyceraldehyde-3-phosphate dehydrogenase (*GAPDH*; NCBI Acc. No. MH260251) was used as an internal control. The PCR program included pre-denaturation at 95 °C for 3 min, followed by 35 cycles at 95 °C for 30 s, 58 °C for 15 s, and 72 °C for 30 s, and final extension at 72 °C for 5 min. Relative gene expression was calculated as described earlier [71,72]. 

### 5.8. Chlorophyll-a Fluorescence 

Chlorophyll-a fluorescence was recorded on days 18, 19, and 20 on fully expanded leaves that had been dark-adapted for 1 h using a pocket PEA (plant efficiency analyzer; Hansatech Instruments Ltd., King’s Lynn, Norfolk, UK). Chlorophyll-a fluorescence readings from well-watered (WW) plants were used as controls. Actinic light (3000 μmol photons m^−2^ s^−1^) was used for fluorescence induction and fluorescence was recorded at 685 nm. Three leaves were randomly selected from each of the triplicate pots for each treatment. The maximal fluorescence (Fm) and the minimal fluorescence (F_0_) of sampled leaves were used to calculate the Fv/Fm ratio, which is related to the maximal quantum yield of PSII photochemistry [27]. The fluorescence readings were further analyzed using the Biolyzer program (http://www.fluoromatics.com/biolyzer_software-1.php), which calculates different photosynthetic parameters according to the JIP test equations based on the theory of energy fluxes in biomembranes [48]. The formulae and definitions of the terms used in the JIP test are listed in Table S2. 

### 5.9. Statistical Analysis

The experiments in this study were repeated at least three times and the data obtained from different experiments were analyzed using Origin 8.1 software (www.originlab.com). Statistical differences were determined using one-way analysis of variance followed by Tukey’s multiple comparison tests. Standard error was calculated using the *n* values for each experiment. Bars with different letters in the figures indicate significant differences at *p* < 0.05.

## Figures and Tables

**Table 1:** Effect of pre-treatment with melatonin on the growth parameters and chlorophyll (Chl) content on a dry weight basis (dwb) of oat plants exposed to salinity stress.

	Shoot Length (cm)	Root Length (cm)	F.W. (g)	Chl A (mg/g dwb)	Chl B (mg/g dwb)	Total Chl (mg/g dwb)
**WW**	27.5 (±0.95)^a^	14.5 (±0.45)^a^	0.61 (±0.04)^a^	5.32 (±0.12)^a^	7.43 (±0.07)^a^	12.75 (±0.20)^a^
**NaCl**	12.57 (±0.21)^b^	6.21 (±0.1)^b^	0.16 (±0.06)^b^	2.93 (±0.02)^b^	4.72 (±0.06)^b^	7.55 (±0.08)^b^
**Mel+W**	20.65 (±0.1)^c^	10.45 (±0.13)^c^	0.48 (±0.07)^c^	4.47 (±0.02)^c^	7.56 (±0.02)^a^	12.02 (±0.03)^a^
**Mel+NaCl**	15.07 (±0.08)^d^	8.6 (±0.07)^d^	0.24 (±0.04)^d^	3.39 (±0.13)^d^	5.46 (±0.19)^c^	8.84 (±0.33)^c^

WW, well-watered oat plants (control); NaCl, oat plants exposed to 300 mM NaCl for 10 days; Mel+W, oat plants exposed to melatonin for 7 days then watered normally for 10 days; Mel+NaCl, plants treated with melatonin for 7 days then exposed to 300 mM NaCl for 10 days. Values are the mean ± SE of three independent assays of four replicates in each treatment. Different letters in each column indicate significant differences (*p* ≤ 0.05) between treatments; Tukey’s test (*n* = 4).

## Supplementary Materials

The following are available online at https://www.mdpi.com/2223-7747/8/12/610/s1. TableS1: Primers used in the study, Table S2. Formulae and glossary of terms used by the JIP-test for the analysis of Chl a fluorescence transient OJIP emitted by dark-adapted photosynthetic samples.
